# Inhibition of DNA Gyrase by Levofloxacin and Related Fluorine-Containing Heterocyclic Compounds

**Published:** 2011

**Authors:** V.L. Tunitskaya, A.R. Khomutov, S.N. Kochetkov, S.K. Kotovskaya, V.N. Charushin

**Affiliations:** Engelhardt Institute of Molecular Biology, Russian Academy of Sciences; Postovsky Institute of Organic Synthesis, Ural Branch, Russian Academy of Sciences; Urals Federal University, Yekaterinburg, Russia

**Keywords:** fluoroquinolones, levofloxacin, derivatives, bacterial DNA gyrase, enzymatic activity, inhibition

## Abstract

Fluoroquinolones are an important class of modern and efficient antibacterial drugs with a broad spectrum of activity. Levofloxacin (the optically active form of ofloxacin) is one of the most promising fluoroquinolone drugs, and its antibacterial activity is substantially higher than the activity of other drugs of the fluoroquinolone family. Earlier, in the Postovsky Institute of Organic Synthesis, UB RAS, an original method of levofloxacin synthesis was developed, and now the pilot batch of the drug is being prepared. Bacterial DNA gyrase is a specific target of fluoroquinolones; hence, the study of the enzyme-drug interaction is of theoretical and practical importance. Moreover, the parameters of DNA gyrase inhibition may serve as a criterion for drug quality. Here, we present the results of studying the interaction of DNA gyrase with a number of fluoroquinolones and their analogs: intermediates and semi-products of the levofloxacin synthesis, and also samples from the pilot batches of this drug. The importance of two structural elements of the levofloxacin molecule for the efficiency of the inhibition is revealed. The data obtained may be useful for the design of new drugs derived from levofloxacin.

## INTRODUCTION

Fluoroquinolones are among the most important classes of effective antibacterial drugs with a broad spectrum of activity. They effectively compete with and can partly substitute cephalosporins and other antibio­tics, which are widely used in clinical practice to cure infectious diseases [1–11]. The first fluoroquinolones (pefloxacin, ciprofloxacin, norfloxacin, and ofloxacin) appeared in the global pharmaceutical market in the early 1990s [1–6]. Now, sales of ciprofloxacin amount to about 10 billion US dollars, and novel promising drugs of this family, such as levofloxacin [12] (one of the enantiomers of ofloxacin) and moxifloxacin, have been developed. Therefore, fluoroquinolones occupy an important place among the arsenal of antibiotics currently in use.

Fluoroquinolones are effective against a large number of diseases: severe suppurative-septic infections, including those of the respiratory tract, urinary tract, skin and soft tissues; bones and joints; liver and billiary; gastrointestinal tract, eyes and the central nervous system; and sexually transmitted infections [1–4]. The high efficiency of fluoroquinolones and wide spectrum of antibacterial activity is due to their ability to affect the reproduction of bacteria inhibiting bacterial topoisomerase II (DNA gyrase), an enzyme responsible for the breaking and restoration of the DNA double helix. It is of importance that the mechanism of fluoroquinolone action differs from that of the other groups of antibiotics (e. g., penicillin antibiotics, cephalosporins, and aminoglycosides), which enables the effective use of fluoroquinolones for the treatment of infectious diseases caused by antibiotic-resistant strains [1–4].


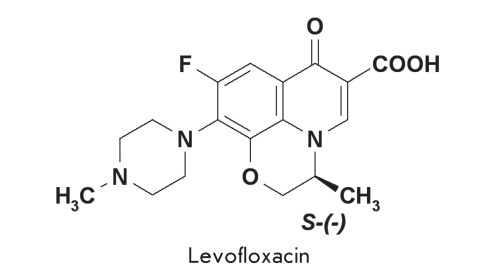
Levofloxacin

One of the most effective drugs from the group of tricyclic fluoroquinolones is levofloxacin, which is an optically active from ( *S* -isomer) of ofloxacin. The antibacterial activity of levofloxacin is twice higher than the activity of racemic ofloxacin and 128 times higher than the activity of its *R* -antipode [13]. Levofloxacin is rightly referred to as a drug of the 21st century. This drug, at low doses, affects clinically important gram-positive and atypical microorganisms. Besides, it exhi­bits high activity towards many gram-negative bacteria [12]. Original methods for the preparation of a family of fluoroquinolones and the corresponding synthetic precursors, including enantiomerically pure semi-products of levofloxacin synthesis, based on kinetic separation of optical antipodes using chiral reagents were recently developed at the Postovsky Institute of Organic Synthesis, UB RAS, and the Urals Federal University [5, 7, 11]. The impact of these studies, mainly the synthesis of optically active levofloxacin and its analogs, has been confirmed by a series of publications [14–20], including an original and effective method for the production of ( *S* )-7,8-difluoro-2,3-dihydro-3-methyl-4H-[1,4]-benzoxazine, which is a key semi-product of levofloxacin synthesis [14, 15]. 

Currently, in the Postovsky Institute of Organic Synthesis, UB RAS, the design and synthesis of new fluoroquinolone derivatives and their analogs with antibacterial activity are in progress. An important constituent of these studies is the investigation of the interaction of new compounds with DNA gyrase, which may be considered among the criterion for the purity of levofloxacin. In this paper, we present results of the investigation of the interaction of DNA gyrase with new fluoroquinolone derivatives, as well as with levofloxacin samples from the pilot batch.

## EXPERIMENTAL

**Materials**

 GyrA-pET19 and GyrB-pET19m plasmids containing genes encoding the A and B subunits of DNA gyrase were kindly provided by K.V. Severinov and I.S. Shkundina (Institute of Molecular Genetics, RAS) and were used as producers of DNA gyrase. The substrates for the gyrase reaction (i. e., a relaxed plasmid pBR322 or pHOT) were purchased from Topogen (USA). 

**Isolation and Purification of DNA Gyrase**

*Escherichia coli *Rosetta (DE3) { *F- ompT hsdSB (rbmB-)gal dcm lacY1 (DE3) pRARE6 (CmR)* } (“Novagen”, USA) was used as the expression strain. The DNA gyrase subunits encoded by the plasmids contained six histidine residues at the *N* -terminus; the latter facilitated their isolation with affinity chromatography using Ni-NTA-agarose. 

The cells transformed with plasmids were grown overnight in 5 ml of the LB medium containing 150 mg/l ampicillin (A150) and 15 mg/l chloramphenicol (C15) at 37°C. The cell pellet was obtained by centrifugation and finally re-suspended in 250 ml of the fresh medium containing A150 and C15 and grown up to an optical density (OD _550_ ) of 0.5 at 37°C. Then, isopropylthio-β- *D* -galactoside (IPTG) was added (final concentration 1 mM), and cultivation was continued for an additional 18 h at 17°C. Cells were collected by centrifugation at 4000 rpm for 20 min, washed with a GTE buffer (25 mM Tris-HCl, pH 7.6, 50 mM glucose, and 10 mM EDTA), and kept frozen at –85°C. Furthermore, the cells were suspended in 15 ml of buffer A (20 mM Tris-HCl, pH 8.0, 500 mM NaCl, 10% (v/v) glycerol, 1% (v/v) Triton X-100, and 1 mM 2-mercaptoethanol), and protease inhibitors (1 mM phenylmethylsulfonyl fluoride (PMSF) and 10 µg/ml aprotinin) were added. The suspension of cells was sonicated on ice and centrifuged for 15 min at 10000 g. The supernatant was applied on the column with Ni-NTA-agarose (2 ml) equilibrated with buffer A. The column was subsequently washed with buffer A containing 10, 30, and 50 mM of imidazole (5 ml each), and the target proteins were eluted with the same buffer containing 200 mM of imidazole. The fractions containing gyrase A and gyrase B were dialyzed against buffer A without Triton X-100 and then against the same buffer containing 50% of glycerol. The yield and the purity of the enzymes were analyzed by 12% polyacrylamide gel electrophoresis in accordance with the procedure described by Laemmli. The yield of gyrase A was 40 mg/l of the cell culture; gyrase B, around 60 mg/l. Equivalent amounts of the thus-obtained DNA gyrase subunits A and B were mixed, and the ali­quots at a volume of 30–50 µl could be stored at –80°C for several weeks without a significant loss of activity, while these enzyme samples remained active for only 2–3 days at –18° C. 

**Determination of the Activity of DNA Gyrase**

**Fig. 1:**
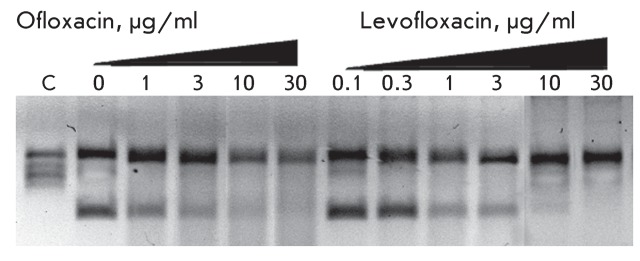
Inhibition of DNA gyrase by levofloxacin and ofloxacin. Figures specify the concentrations of inhibitors in the reaction mixtures. C – control (plasmid without the enzyme).

**Fig. 2:**
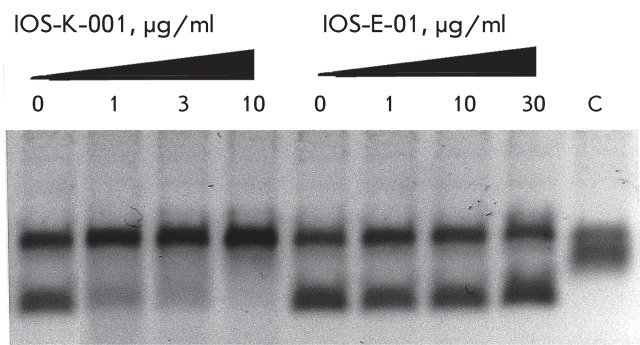
Inhibition of DNAgyrase by IOS-K-001 and IOS-E-01. Figures specify the concentrations of inhibitors in the reaction mixtures. C – control (plasmid without the enzyme). Reaction conditions are described under “Experimental.”

 The reaction mixture (30 µl) contained 35 mM Tris-HCl (pH 7.5), 24 mM KCl, 4 mM MgCl _2_ , 1.4 mM ATP, 5 mM DTT, 1.8 mM spermidine, and 0.1 mg/ml of a bovine serum albumin. A mixture of DNA gyrase A and B subunits (0.4 µg) and 0.25–0.5 µg of the substrate (relaxed plasmid pBR322) were added to the reaction probe. The reaction was performed for 60 min at 25°C (for multiple samples, 96-well plates were used). After the reaction was completed, the samples were extracted with an equal volume of chloroform (30 µl). Then, 7 µl of the mixture containing 50% of sodium dodecyl sulfate, 25% of glycerol, and 0.25% of bromphenol blue were added to the aqueous phase. The mixture was applied on a 1.2% agarose gel in TAE buffer (40 mM Tris-acetate, pH 8.0, 2 mM EDTA) and analyzed with electrophoresis (50 V, 2 h). The products were visualized under UV-light after staining with ethidium bromide. The activity of the enzyme (%) was determined from the intensity of the band corresponding to the super-coiled form of plasmid pBR322 and estimated using the Total Lab v2.01 software. 

**Inhibition of DNA Gyrase by Fluoroquinolones and Related Fluorine-Containing Heterocycles**

 The reaction mixture in a volume of 30 µl contained 35 mM Tris-HCl (pH 7.5), 24 mM KCl, 4 mM MgCl _2_ , 1.4 mM ATP, 5 mM DTT, 1.8 mM spermidine, 0.1 mg/ml bovine serum albumin, and a solution of levofloxacin or fluorine-containing heterocyclic derivatives (see below) in DMSO (1-3 µl). An equal volume of DMSO was added to the control. The reaction was started upon addition of DNA gyrase (0.4 µg) and relaxed plasmid pBR322 (0.25–0.5 µg), which was used as a substrate, and performed as described above. Calculations were made using the Total Lab v2.01 program. 

## RESULTS AND DISCUSSION

The cellular target of fluoroquinolones is bacterial DNA gyrase, which consists of two subunits with M _r_ of 105 and 95 kDa and encoded by the *gyrA* and *gyrB* genes, respectively. DNA gyrase has an A _2_ B _2_ -type tetrameric structure [1–5]. Although this enzyme does not only break but also ligate the breaks in the DNA chains, normally, only one reaction is used to determine the enzyme activity *in vitro* ; namely, the ability of the enzyme to produce a negative super coil form of the plasmid from the relaxed circular DNA. Respectively, the substrate and the product can be separated and determined quantitatively using agarose gel electrophoresis. 

The DNA gyrase from * E. coli* , which was used in this work, had high specific activity that enabled us to use these enzyme samples for testing the inhibitory activity of fluoroquinolones. First, levofloxacin (a left-rotating isomer of ofloxacin) with *ee* of about 99% was studied using ofloxacin (racemic form) as a control. Ofloxacin is well-known to clinicians as a fluoroquinolone of the first generation that has been in use in practical therapy for more than 15 years [1–4]. 

**Fig. 3:**
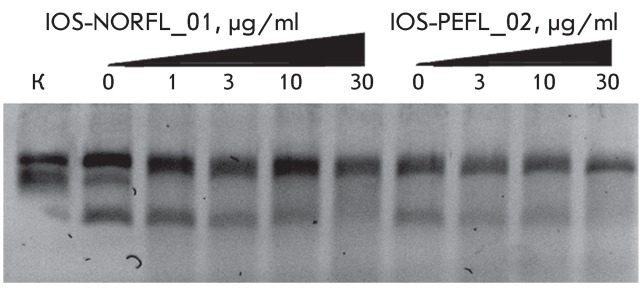
Inhibition of DNA-gyrase by norfloxacin IOS-NORFL_01 and pefloxacin IOS-PEFL_02. Figures specify the concentrations of inhibitors in the reaction mixtures. C – control (plasmid without the enzyme). Reaction conditions are described under “Experimental.”

**Table 1:** Structural formulas of levofloxacin, intermediates, and semi-products of its synthesis

Structural formula	Substance	Type of the substance	Code
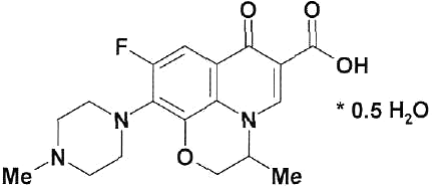	Levofloxacin(3*S*)-9-Fluoro-3-methyl-10-(4-methyl piperazine-1-il)-7-oxo-2,3-dihydro-7H-pyrido[1,2,3-*d,e*]-benzoxazine-6-carboxylic acid hemihydrate	Substance	IOS-001 – IOS-019
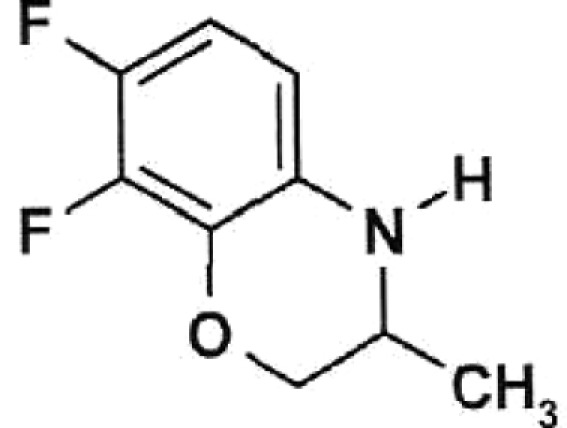	(*R,S*)-7,8-Difluoro-3-methyl-2,3-dihydro-4H-benzo[*b*][1,4]-oxazine	Intermediate	IOS-RS-01, IOS-RS-02
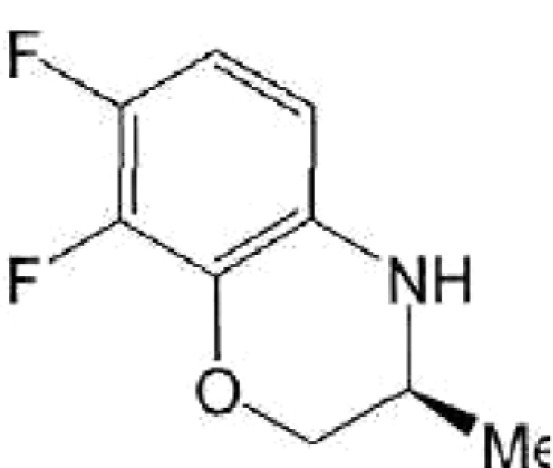	(3*S*)-2,3-Dihydro-3-methyl-7,8-difluoro-1,4-benzoxazine	Intermediate	IOS-S-01, IOS-S-02
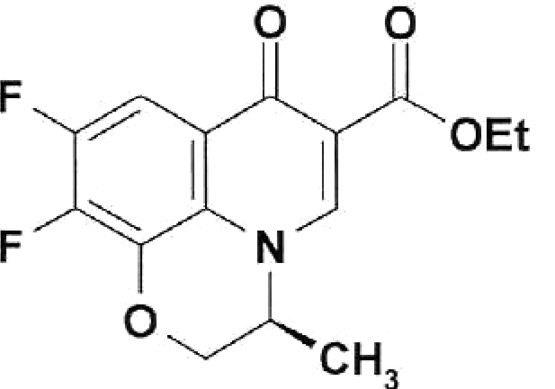	Ethyl ester of (3*S*)-(-)-9,10-difluoro-3-methyl-7-oxo-2,3-dihydro-7H-pyrido[1,2,3-*d,e*][1,4]-benzoxazine-6-carboxylic acid	Semi-product	IOS-Е-01,IOS-Е-02
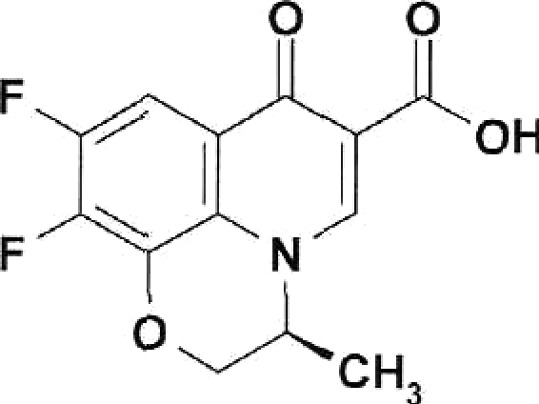	(3*S*)-(-)-Difluoro-3-methyl-7-oxo-2,3-dihydro-7H-pyrido[1,2,3-*d,e*][1,4]-benzoxazine-6-carboxylic acid	Semi-product	IOS-К-001,IOS-К-002

Inhibition of DNA gyrase by the above fluoroquinolones are presented in *Fig. 1* . The IC _50_ values calculated from the results of three independent experiments were 2.50 ± 0.14 µg/ml and 6.20 ± 0.17 µg/ml for levofloxacin and ofloxacin, respectively, and they are in general agreement with the published data [7–10]. Moreover, the IC _50_ ratios for these substances indicate the stereospecificity of DNA gyrase. The IC _50_ value for the racemate (ofloxacin) is approximately twice higher than that for the individual stereoisomer (levofloxacin), which is roughly equal to its content in the racemic mixture. 

Since the IC _50_ value is widely used to describe the efficiency of the inhibition, it can be used to control the quality of levofloxacin. Taking into account that only one isomer of ofloxacin efficiently inhibited the enzyme, one can also consider the IC _50_ value as a criterion of drug purity. Hence, biological activity, as well as chemical purity and the *ee* value, is considered to be an important characteristic of the quality of levofloxacin. 

The structural formulas of levofloxacin, the key intermediates and semi-products of its pilot-scale synthesis preformed in the Postovsky Institute of Organic Synthesis, UB RAS, are presented in *Table 1.*

All 19 samples of the levofloxacin taken from the pilot batch had close IC _50_ values (2.4–2.8 µg/ml); i.e., they are in close correlation, since the error of the methods used is about 20%, and they are in agreement with the data published for the sample of levofloxacin of 99% purity. Among the key intermediates and semi-products of levofloxacin synthesis, racemic benzoxazine ( **IOS-RS** ), enantiomerically pure benzoxazine ( **IOS-S** ), and the ester ( **IOS-E** ) exhibited no significant activity. The activity of the semi-product **IOS-K** was found to be lower by an order of magnitude as compared with that of levofloxacin ( *Fig. 2* ). 

**Table 2:** Inhibition activity of a series of fluoroquinolones and fluorine-containing heterocycles towards DNA gyrase

CODE/I50 µg/ml	Structural formula	CODE/I50 µg/ml	Structural formula
EV-465 >30	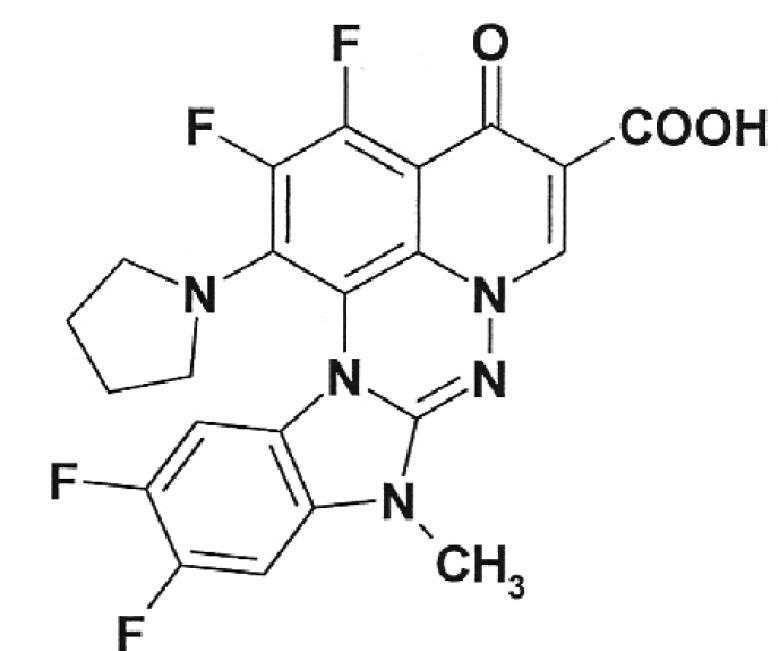	EV-X149 Inactive	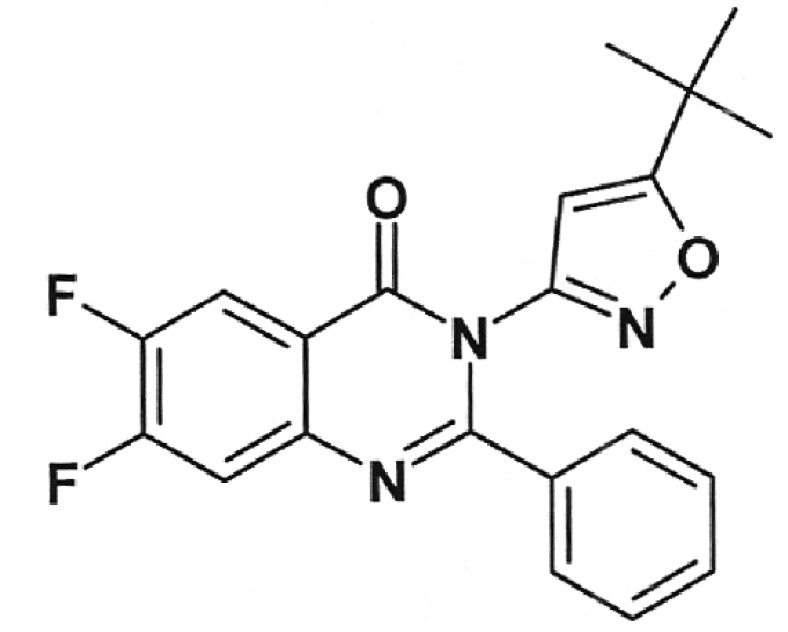
EV-591 Inactive	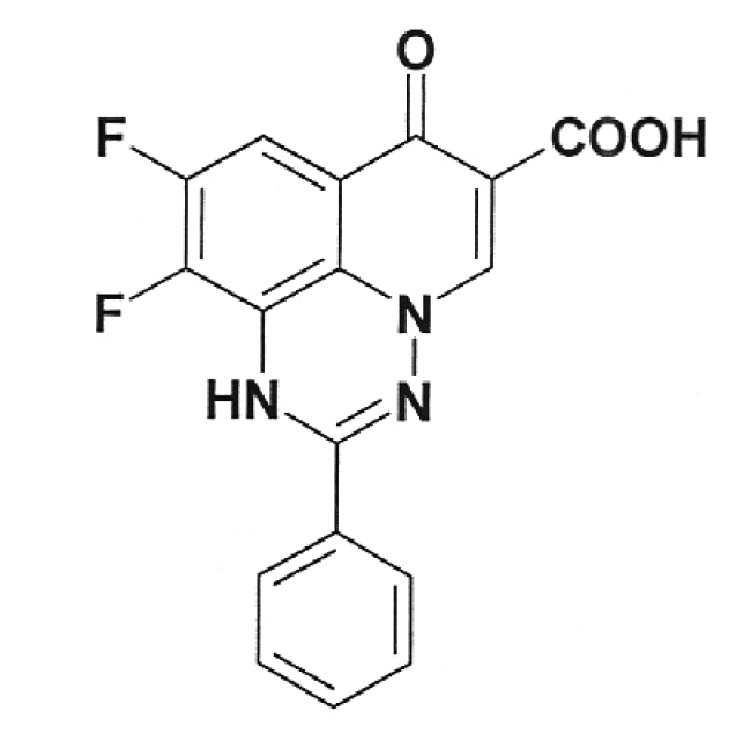	EV-T150-C Inactive	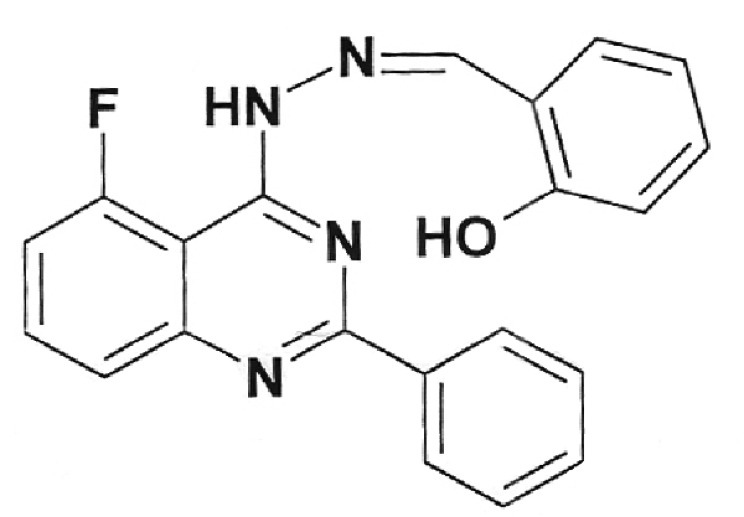
EV-452-D Inactive	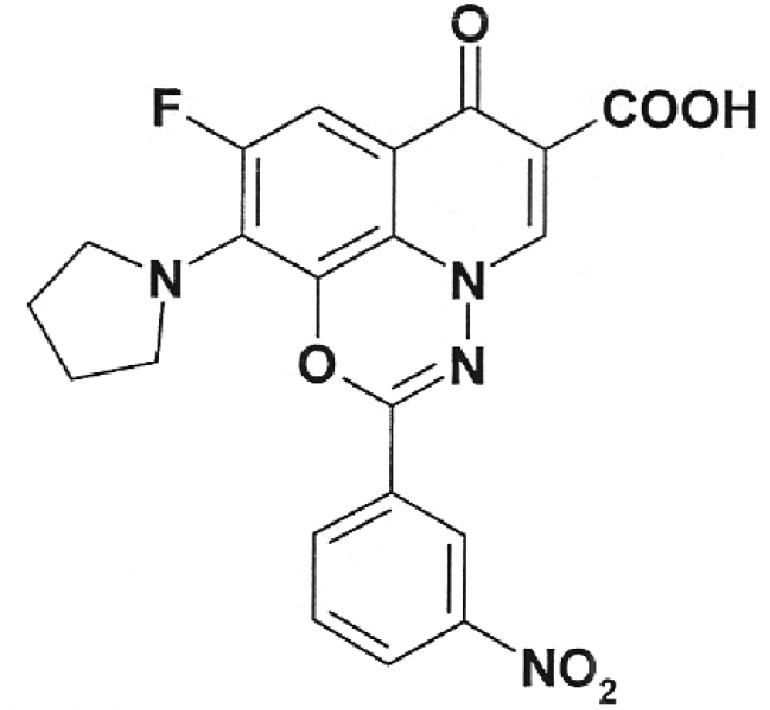	EV-T156 Inactive	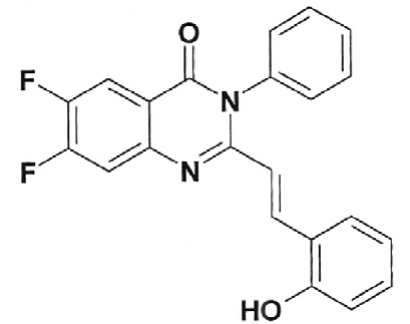
EV-313-D Inactive	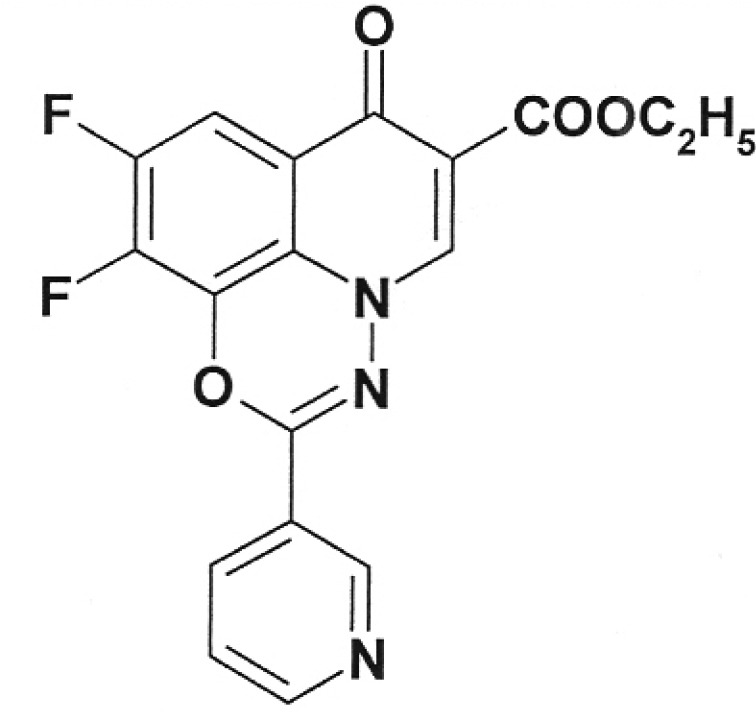	EV-T143-B Inactive	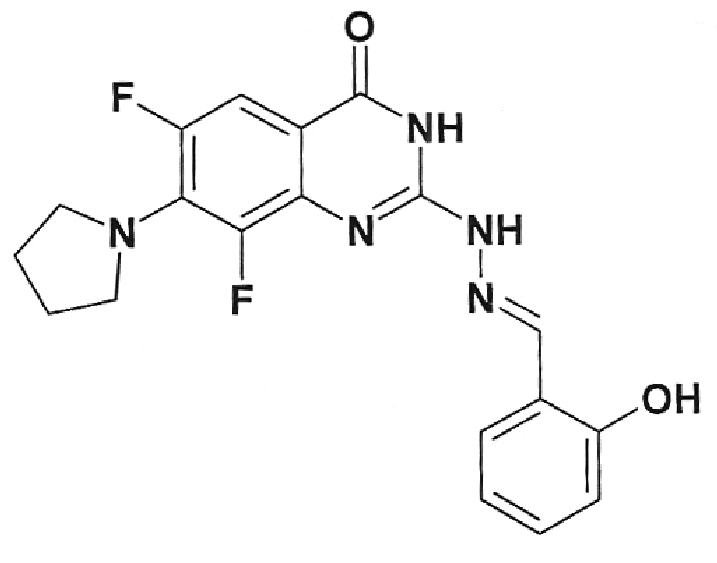
EV-572 Inactive	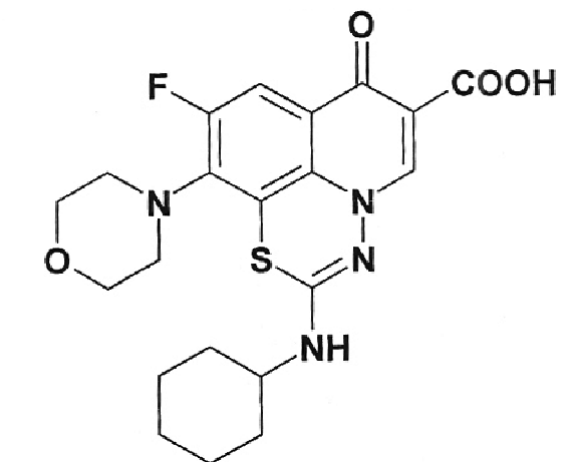	EV-N51-B Inactive	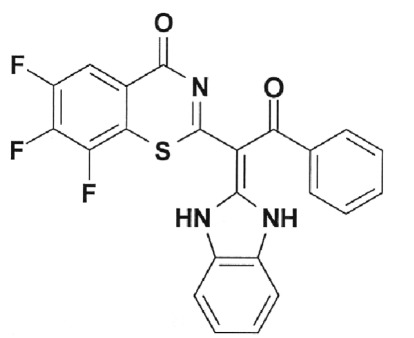
EV-X58-A Inactive	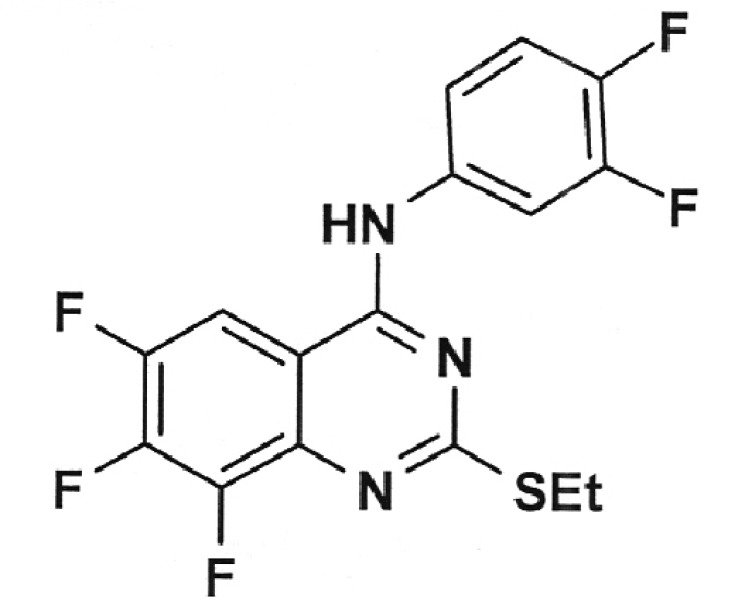	EV-N119-2 Inactive	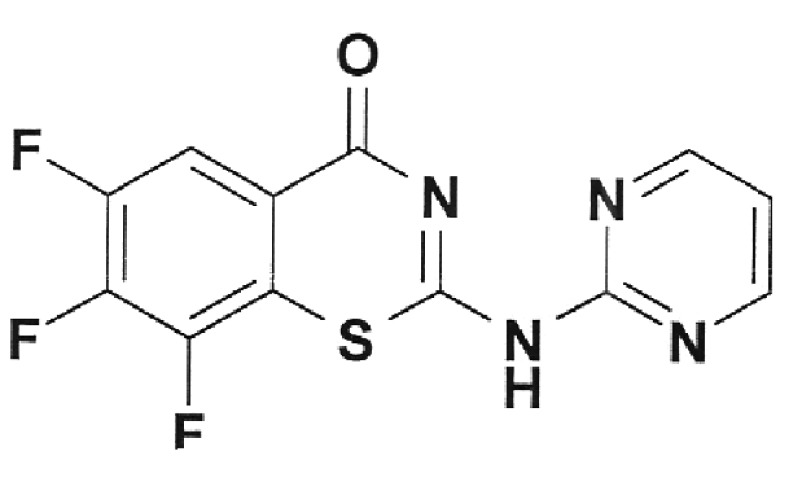
IOS-NORFL_01>11	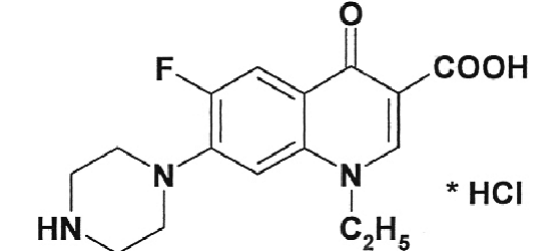	IOS-PEFL_02 2.8	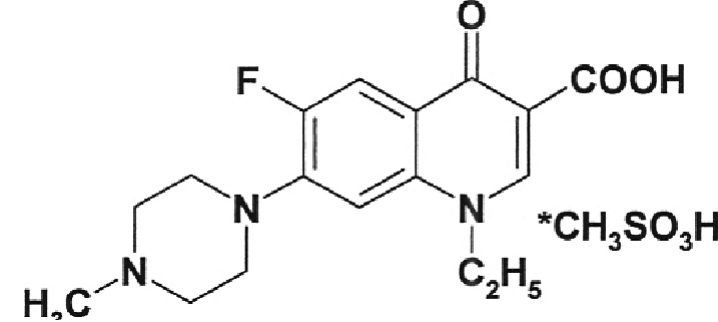

**Fig. 4:**
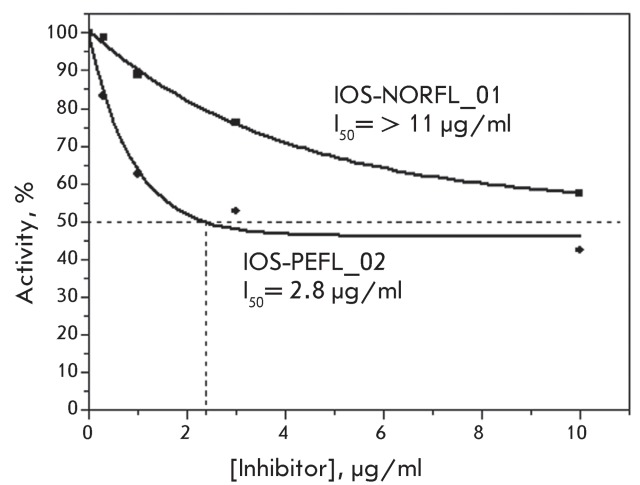
Determination of the I _50 _ value for the compounds IOS-NORFL_01 and IOS-PEFL_02 (from the data shown in Fig. 3). The I _50 _ value corresponds to the point at which dash lines intersect the X-axis.

The comparison of the structural formulas for the semi-products **IOS-E** and **IOS-K ** reveals that these samples differ only by the ethoxycarbonyl group in **IOS-E, ** instead of the free carboxyl group in **IOS-K** . It is important to note that this difference is crucial for the activity; i. e., the substance possessing the carboxyl group shows significant inhibitory activity, while the ethoxycarbonyl derivative is practically inactive. This observation indicates the significant impact of the charged carboxyl group in the inhibition. These data are in accordance with the known mechanism of the interaction of fluoroquinolones with DNA gyrase and should be taken into account when designing novel and more effective inhibitors of the enzyme. 

In this paper, the ability of several new fluoroquinolone derivatives and fluorine-containing heterocycles to inhibit DNA gyrase was also investigated. These substances were synthesized in the Postovsky Institute of Organic Synthesis, UB RAS. The results of the experiments demonstrated that the majority of the studied compounds are inactive as depicted in *Table 2* . The only exceptions is fluoroquinolone **EV-465** , the activity of which is lower than the activity of levofloxacin by approximately for two orders of magnitude and the known compounds from the fluoroquinolone group, i.e. **IOS-NORFL_01** (norfloxacin) and **IOS-PEFL_02** (pefloxacin) ( *Figs. 3, 4* ). 

The compound ** IOS-NORFL_01** turned out to be a poor inhibitor, while substance **IOS-PEFL_02** showed moderate activity, which was significantly lower than the activity of levofloxacin. It is important to note that these compounds differ only by the presence of an additional methyl group in the piperazine ring of **IOS-PEFL_02** . The introduction of the methyl group results in an increase in the efficiency of the inhibition by approximately an order of magnitude, and this observation is of importance for the future design of novel biologically active fluoroquinolones. 

The data obtained confirm that the degree of inhibition of bacterial DNA gyrase by levofloxacin and other fluoroquinolones is an important addition to physicochemical methods, when the quality of the synthesized drugs is controlled. Using this method, the quality of 19 samples from the pilot batch of levofloxacin was assessed, along with eight intermediates and semi-products, as well as 14 new fluoroquinolone derivatives and their analogs.

Structure-activity relationship data outlined the importance of the carboxyl group in the **IOS-K** structure, the etherification of which leads to the loss of the inhibitory activity, and the N-methyl group in compound **IOS-PEFL** , which is also essential for the activity. The data obtained should be considered during the design of novel drugs based on fluorine-containing heterocycles. 

## References

[1] Eds Siporin C., Heifetz C.L., Domagala J.M. (1990). The New Generation of Quinolones.

[2] Shen L.L. (1993). Quinolone Antibacterial Agents. Soc. Microbiol..

[3] Eds Hooper D.S., Wolfson J.S. (1993). Quinolone Antimicrobial Agents.

[4] Padeyskaya E.N., Yakovlev V.P. (1995). Fluoroquinolones Moscow: Bioinform,.

[5] Mokrushina G.A., Charushin V.N., Chupakhin O.N. (1995). Pharmaceutical Chemistry Journal.

[6] Andriole T.V. (1998). Ball P. The Quinolones. Acad. Press.

[7] Mokrushina G.A., Nosova E.N., Lipunova G.N., Charushin V.N. Russian Journal of Organic Chemistry.

[8] Granik V.G. (2001). Basic Medicinal Chemistry Moscow: Vuzovskaya Kniga,.

[9] Furin G.G. (2001). Fluorine-containing Heterocyclic compounds: Synthesis and Applications
Novosibirsk:Nauka,.

[10] Mokrushin V.P., Vavilov G.A. (2004). Principles of the Chemistry and Technology of bioorganic and synthetic
medical preparations. Ekaterinburg:UGTU-UPI,.

[11] Nosova E.V., Mochulskaya N.N., Kotovskaya S.K., Lipunova G.N., Charushin V.N. (2006). Heteroatom. Chemistry..

[12] Yakovlev S.V. Infections and Antimicrobial Therapy.

[13] Furukawa M. (1986). Antimicrob. Agents Chemother..

[14] Krasnov V.P., Levit G.L., Korolyova M.A., Kodess M.I., Chupakhin O.N., Kim M.H., Lee H.S., Park Y.J., Kim K.-C. (1999). Tetrahedron: Asymmetry,.

[15] Chupakhin O.N., Krasnov V.P., Levit G.L., Charushin V.N., Korolyova M.A., Tzoi E.V., Lee H.S., Park Y.J., Kim M.H., Kim K.Ch. (2000). Japanese Patent JP 2000178265. Production of (S)-benzoxazine derivative
and racemization of (R)-benzoxazine derivative.

[16] Krasnov V.P., Levit G.L., Bukrina I.M., Andreeva I.N., Sadretdinova L.Sh., Korolyova M.A., Kodess M.I., Charushin V.N., Chupakhin O.N. (2003). Tetrahedron: Asymmetry,.

[17] Potemkin V.A., Krasnov V.P., Levit G.L., Bartashevich E.V., Andreeva I.N., Kuzminsky M.B., Anikin N.A., Charushin V.N., Chupakhin O.N. (2004). Mendeleev Comm..

[18] Krasnov V.P., Levit G.L., Kodess M.I., Charushin V.N., Chupakhin O.N. (2004). Tetrahedron: Asymmetry..

[19] Gruzdev D.A., Levit G.L., Krasnov V.P., Chulakov E.N., Sadretdinova L.Sh., Grishakov A.N., Ezhikova M.A., Kodess M.I., Charushin V.N. (2010). Tetrahedron: Asymmetry..

[20] Levit G.L., Gruzdev D.A., Krasnov V.P., Chulakov E.N., Sadretdinova L.Sh., Ezhikova M.A., Kodess M.I., Charushin V.N. (2011). Tetrahedron: Asymmetry..

